# Is *Candida auris* the first multidrug-resistant fungal zoonosis emerging from climate change?

**DOI:** 10.1128/mbio.00146-24

**Published:** 2024-03-13

**Authors:** Victor Garcia-Bustos

**Affiliations:** 1 Severe Infection Research Group, Health Research Institute La Fe, Valencia, Spain; 2 Instituto Universitario de Sanidad Animal y Seguridad Alimentaria (IUSA), Universidad de Las Palmas de Gran Canaria, Arucas, Spain; 3 Department of Internal Medicine and Infectious Diseases, University and Polytechnic Hospital La Fe, Valencia, Spain; Duke University Hospital, Durham, North Carolina, USA

**Keywords:** *Candida auris*, zoonosis, dog, animal, thermal resistance, climate change

## Abstract

The emergence and evolutionary path of *Candida* auris poses an intriguing scientific enigma. Its isolation from a pet dog’s oral cavity in Kansas, reported by White et al. (T. C. White, B. D. Esquivel, E. M. Rouse Salcido, A. M. Schweiker, et al., mBio 15:e03080-23, 2024, https://doi.org/10.1128/mbio.03080-23), carries significant implications. This discovery intensifies concerns about its hypothetical capacity for zoonotic transmission, particularly considering the dog’s extensive human contact and the absence of secondary animal/human cases in both animals and humans. The findings challenge established notions of *C. auris* transmissibility and underscore the need for further investigation into the transmission dynamics, especially zooanthroponotic pathways. It raises concerns about its adaptability in different hosts and environments, highlighting potential role of environmental and animal reservoirs in its dissemination. Critical points include the evolving thermal tolerance and the genetic divergence in the isolate. This case exemplifies the necessity for an integrated One Health approach, combining human, animal, and environmental health perspectives, to unravel the complexities of *C. auris*’s emergence and behavior.

## COMMENTARY

*Candida auris* is an emerging fungal pathogen distinguished by its unprecedented multidrug resistance, environmental survivability, and high transmissibility. Its evolutionary trajectory presents a significant and intriguing scientific enigma. Whole-genome sequencing has identified five genetically distinct clades that appeared nearly simultaneously and independently across three continents (1, 2). Population genomics dates the most recent common ancestor of each clade to within the last 360 years. Notably, the clusters causing outbreaks in clades I, III, and IV are thought to have emerged 36 to 38 years ago, suggesting a recent divergence and the acquisition of virulence from a non-pathogenic environmental ancestor (3). This unusual pattern of emergence challenges traditional epidemiological models and indicates that a founder effect may have played a crucial role in the global spread and independent evolutionary paths of *C. auris*, predating its recognition in clinical environments.

Climate change and animal colonization are hypothesized to play roles in the evolutionary dynamics of *C. auris* (4, 5). The 2019 study by Casadevall et al. indicated that *C. auris* possesses greater thermal tolerance than its marine relatives, such as *C. haemulonii*. Furthermore, an important saline tolerance has also been reported in several studies, demonstrating enhanced resistance to salinity compared to other *Candida* species, such as *C. albicans* (6, 7). This enhanced halotolerance and thermal adaptability suggest its origin in saline ecosystems like wetlands and its subsequent evolution into a pathogen or colonizer in endothermic species, potentially aiding its global dissemination (4). The isolation of *C. auris* from environmental sources in the Andaman Islands in 2021 and Colombian estuaries in 2022 confirmed its presence in marine habitats (6, 8). Additionally, its detection in public swimming pools in the Netherlands in 2018 (9), and hospital and municipal wastewaters in Nevada and Florida in 2022 (10, 11), demonstrates its adaptability to various aquatic environments. A strain from the Andaman Islands, characterized by reduced drug resistance and thermal tolerance (6), appears more closely related to the ancestral marine forms of the fungus, providing insights into its evolution from an environmental organism to a human pathogen. However, animals might play a crucial role in its dissemination.

Initial evidence of *C. auris* in animal hosts emerged from retrospective *in silico* analyses using culture-independent metabarcoding techniques (12). *Candida auris* sequences were identified in the ear canal of a Spanish dog with otitis externa and on the skin of two newt species in Cambridgeshire, UK. Subsequent findings extended beyond metabarcoding evidence; live *C. auris* was isolated from two stray dogs with otitis externa in India (13) and from the oral cavity of a pet dog in Kansas as documented in the interesting study by White et al. (14).

These findings raise critical questions about the hypothetic zoonotic potential of *C. auris*, particularly in the context of climate change. Transmission routes remain to be elucidated, with no documented interspecies spread in the cases from the US and India. The case in Kansas is particularly noteworthy due to the pet dog’s frequent human interaction, contrasting with the established transmissibility of *C. auris* among humans in clinical settings. This instance was the first documented occurrence in Kansas, in an area without previous reports of the pathogen. Notably, despite *C. auris’*s known transmission capability and surface survivability, there were no secondary cases in other dogs at the shelter, including the dog’s littermate, even with regular oral contact among dogs and humans (14). This pattern was also seen in the Indian stray dogs (13).

The absence of screening for *C. auris* in the shelter environment, caregivers, and current owners represents a significant oversight, given the relevance of the finding. However, this contact tracing was performed in the Indian animal intensive care unit, but no colonization in healthcare workers or surfaces was observed. It is yet to be elucidated whether the transmissibility of *C. auris* is altered in colonization of non-human hosts with higher basal temperatures. However, as in humans, the unifocal colonization and the low fungal burden in described cases might also be implicated, especially considering the spontaneous clearance in the Kansas dog.

Moreover, these findings suggest a plausible scenario of zooanthroponotic transmission, where humans previously infected or colonized with *C. auris* could transfer the pathogen to their pets. However, this has not been yet demonstrated. This mode of transmission could establish animals as reservoirs for the fungus, potentially leading to persistent colonization and an expanded host range. Such dynamics might contribute to the acquisition of increased drug resistance or even recurrent candidemia episodes, as previously documented (15).

The ability of *C. auris* to thrive in hosts with basal temperatures exceeding 38°C underscores its ongoing thermal tolerance, a point notably emphasized by White et al. This evolving thermoresistance prompts questions about the role of heat shock proteins and related pathways in enhancing this capacity and its correlation with its virulence, including the potential for increased pathogenicity in these isolates (16). Additionally, the discovery of over 90 single-nucleotide polymorphisms differentiating the clade IV *C. auris* isolate from a Kansas dog from other clade IV strains indicates substantial evolutionary divergence. This genetic diversity warrants functional exploration, as it could influence the pathogen’s characteristics, including virulence, drug resistance, and host range. There is also a notable mention of genetically distinct clade I *C. auris* strains in two Indian dogs. One strain is more closely related to the azole-susceptible Pakistan clade I reference strain, while the other shows a closer relationship to Indian-resistant clinical strains. This variation further exemplifies this pathogen’s genetic complexity and adaptability of this pathogen across different hosts and environments.

These animal isolations underscore that contemporary clinical isolates, including those in animals, have surpassed the thermal barrier traditionally limiting colonization or infection in endothermal hosts. Consequently, we could speculate that a wide range of hosts, including birds with higher basal temperatures and extensive migration patterns involving human contact, could potentially serve as reservoirs for fungal spread. The recent live isolation from dogs adds to the growing body of evidence about *C. auris*’s evolutionary history. Birds have been proposed as possible intermediate hosts in the historical global dissemination of *C. auris* ancestors (4), potentially explaining the founder effect that led to the independent evolution of different clades across continents over the last four centuries. However, the evolutionary leap to bird hosts might be too great for more primitive strains of the fungus (5). Environmental strains are anticipated to be less thermotolerant and drug resistant but more halotolerant compared to the more thermotolerant and multidrug-resistant clinical strains found in animals, such as the amphotericin B-resistant strain isolated from the Kansas dog. An intermediate evolutionary lineage of *C. auris* strains may have historically existed or could still coexist in nature in marine migratory hosts like marine mammals. These hosts, characterized by lower basal temperatures and migration patterns linked to marine ecosystems, could represent a critical evolutionary bridge between environmental and clinical strains.

White et al.’s findings highlight the necessity for a collaborative multifaceted approach, focusing on elucidating transmission dynamics, especially potential zoonotic and zooanthroponotic pathways, and investigating environmental and animal reservoirs. Extensive genomic studies comparing animal, human, and less thermal-tolerant environmental isolates are vital for understanding diversity, evolutionary history, resistance, and virulence. An interdisciplinary One Health approach integrating human, animal, and environmental health perspectives is essential for a comprehensive understanding and management of *C. auris*.
